# Magnetic kyphoplasty: A novel drug delivery system for the spinal column

**DOI:** 10.1371/journal.pone.0201402

**Published:** 2018-07-27

**Authors:** Steven Denyer, Abhiraj D. Bhimani, Steven Papastefan, Pouyan Kheirkhah, Tania Aguilar, Jack Zakrzewski, Clayton L. Rosinski, Akash S. Patel, Saavan Patel, Victoria Zakrzewski, Akop Seksenyan, Gail S. Prins, Ankit I. Mehta

**Affiliations:** 1 Department of Neurosurgery, University of Illinois at Chicago, Chicago, IL, United States; 2 Rosalind Franklin University of Medicine and Science, North Chicago, IL, United States; 3 Department of Urology, University of Illinois at Chicago, Chicago, IL, United States; Laurentian, CANADA

## Abstract

Vertebral compression fractures (VCFs) caused by metastatic malignancies or osteoporosis are devastating injuries with debilitating outcomes for patients. Minimally invasive kyphoplasty is a common procedure used for symptomatic amelioration. However, it fails in treating the underlying etiologies of VCFs. Use of systemic therapy is limited due to low perfusion to the spinal column and systemic toxicity. Localized delivery of drugs to the vertebral column can provide a promising alternative approach. A porcine kyphoplasty model was developed to study the magnetically guided drug delivery of systemically injected magnetic nanoparticles (MNPs). Jamshidi cannulated pedicle needles were placed into the thoracic vertebra and, following inflatable bone tamp expansion, magnetic bone cement was injected to the vertebral body. Histological analysis was performed after intravenous injection of MNPs. Qualitative analysis of harvested tissues revealed successful placement of magnetic cement into the vertebral body. Further quantitative analysis of histological sections of several vertebral bodies demonstrated enhanced accumulation of MNPs to regions that had magnetic cement injected during kyphoplasty compared to those that did not. By modifying the kyphoplasty bone cement to include magnets, thereby providing a guidance stimulus and a localizer, we were successfully able to guide intravenously injected magnetic nanoparticles to the thoracic vertebra. These results demonstrate an in-vivo proof of concept of a novel drug delivery strategy that has the potential to treat the underlying causes of VCFs, in addition to providing symptomatic support.

## Introduction

Vertebral compression fractures (VCFs) are devastating injuries that occur when the strength of the vertebral body is overcome by combined axial and bending forces on the spine [1]. Pathological fractures resulting from spinal malignancies [2] and osteoporosis [3] account for the most common causes of VCFs. Regardless of the etiology, VCFs present with limited mobility and chronic, intractable, and possibly debilitating pain. Chronically, VCFs can progress to the collapse of the vertebral body, resulting in spinal instability and deformity due to kyphosis. Such abnormal curvature of the spine can lead to loss of height, as well as severe loss of mobility and immense physical deconditioning due to inactivity that inevitably leads to impaired social functioning and a significantly reduced quality of life [3].

Minimally invasive kyphoplasty plays a vital role in the management of VCFs. This surgical procedure involves the insertion of an inflatable bone tamp (IBT) into the vertebral body. subsequent inflation restores the vertebral body back to its proper height, while filling the IBT-created cavity with bone cement re-establishes structural support [4]. Thus, kyphoplasty serves as an effective symptomatic treatment option for VCFs that can significantly improve the quality of life for patients. However, kyphoplasty alone does not offer patients any treatment for the underlying causes of their fractures, thus requiring systemic therapeutics for their long-term disease management. Currently treatments for osteoporosis and spinal malignancies are limited due to low degree of blood perfusion in the spine [5, 6] and systemic toxicity due to the accumulation of these drugs in healthy tissue throughout the body [6, 7]. These barriers can be potentially overcome through targeting strategies that can localize therapeutics to the spinal column.

Magnetic nanoparticles (MNPs) possess unique chemical and physical properties which can be harnessed for targeted drug delivery strategies. In addition to being highly responsive to external magnetic fields, they can be functionalized to carry specific drugs, thereby serving as versatile platforms [8, 9]. Tethering of folic acid to MNPs has the advantage of increasing the targeting specificity of chemotherapeutics to cancer cells [10]. Additionally, the ability to modify drug loaded MNPs to be pH and temperature sensitive allows for chemotherapeutics to be released only once they are incorporated at the site of tumor growth [11–14]. This ability decreases the systemic toxicity associated with chemotherapeutics. Indeed, these features of nanoparticles have been previously used to deliver chemotherapeutics [11, 12, 15, 16] and nucleic acids [17] to specific tumor sites while demonstrating decreased systemic drug toxicity and increased treatment efficacy. In addition, drug loaded MNPs demonstrated continued and sustained drug release, which is important for long term management of chronic etiologies associated with VCFs. As such, these multipurpose nanoparticles can serve as ideal carrier platforms for targeting drugs to the spinal column. Furthermore, MNPs are biocompatible and MRI responsive, allowing them to be used therapeutically and monitored by non-invasive imaging modalities [10, 18].

In this study, we show that MNPs can be effectively localized to the spinal column by modifying the kyphoplasty procedure. By adding magnets to the kyphoplasty bone cement, we created a physical stimulus for the localization of the magnetic nanoparticles. This strategy has the potential to serve as a novel and extremely effective drug delivery system that combines both symptomatic and disease-modifying treatments for VCFs.

## Materials and methods

### Experimental design

All experiments involving animals were approved by the Animal Care and Use Committee (ACUC) of the University of Illinois at Chicago and conducted in accordance with the guidelines and regulations of the ACUC. One Landrace-Yorkshire cross pig underwent a kyphoplasty procedure with Polymethyl methacrylate (PMMA) cement and the placement of 12 stacked (each 1/16" dia. x 1/8" thick) neodymium-iron-boron (NdFeB) magnets (K&J Magnetics, Inc.) placed in an upper thoracic vertebral body. Additionally, an internal control of PMMA without magnets was placed two vertebrae levels below the magnet enhanced kyphoplasty. Thus, the pig underwent kyphoplasty at two separate vertebral bodies, with the vertebrae containing magnets being two levels above the PMMA only vertebra.

### Kyphoplasty surgical procedure

A mixture of tiletamine with zolazepam (4.4 mg/kg) and xylazine (2.2 mg/kg) was used as preoperative anesthetic, and then 2% isoflurane in 100% oxygen at 1 L/min maintained anesthesia during surgery. Localization of the proper vertebral bodies was done via C-arm image intensifier X-ray. Once the proper vertebral bodies were identified, the surgical site was prepped and local anesthesia administered. A Jamshidi cannulated needle was inserted through the pedicle and into the vertebral body under X-ray guidance. Inflatable tamps were inserted bilaterally through the injection cannula following removal of the guidewire, and inflated to 200 mmHg, compacting the surrounding bone. 1.5 cc of PMMA cement (with or without magnet) was implanted into each vertebral body while being monitored by anteroposterior and lateral imaging to ensure adequate filling and positioning of the magnets. The PMMA cement was allowed to harden and set before removal of the injection cannula, followed by approximation of the incision with #4–0 nylon sutures, and application of compression dressings.

### Iron core gold coated nanoparticle synthesis

Magnetic nanoparticles used in this study were synthesized according to a previously published technique by Venugopal et al. [19] The magnetic nanoparticle core was synthesized via coprecipitation of ammonium ferric hydroxide and ammonium ferrous hydroxide in sodium hydroxide and given a gold coat by reduction of chloroauric acid. Nanoparticles were collected by first decantation, then thorough washing with nanopure water, followed by 0.2um filtration. The resulting MNPs were recovered by magnetic decantation, filtered (0.2μm filter) and stored at 4°C [20].

### Intravenous injection of magnetic nanoparticles

24-hours after completion of the kyphoplasty, a 50 mg/mm^2^ solution of the MNPs was injected into the right ear vein over 15 minutes under the guidance of a veterinarian.

### Histopathology

24 hours after the nanoparticle injection, the animal was euthanized by intravenous administration of 4.4 mg/kg Telazol and 2.2 mg/kg Zylazine. The thoracic vertebral bodies were harvested, along with a lumbar vertebral body which was not operated on. The vertebrae were grossly imaged, fixed with 4% paraformaldehyde, then prepared for histological processing by decalcification with hydrochloric acid prior to being embedded in paraffin. Continuous 12 μm thick sections were prepared and stained with Prussian blue for iron, and counterstained with nuclear fast red for visualization of structure (IHC world protocol). Ten fields of view at 200x were taken from tissue samples of each of the vertebral bodies. ImageJ was used to quantify the Prussian blue staining in terms of number of pixels and percentage of pixels.

### Statistical analysis

The data were analyzed using GraphPad Prism 7. A one-way ANOVA was used to test for statistical significance. Differences were considered statistically significant if p<0.05. All data are presented as mean ± standard deviation.

## Results

### Establishing a porcine minimally invasive magnetic kyphoplasty model

**Fig 1:**
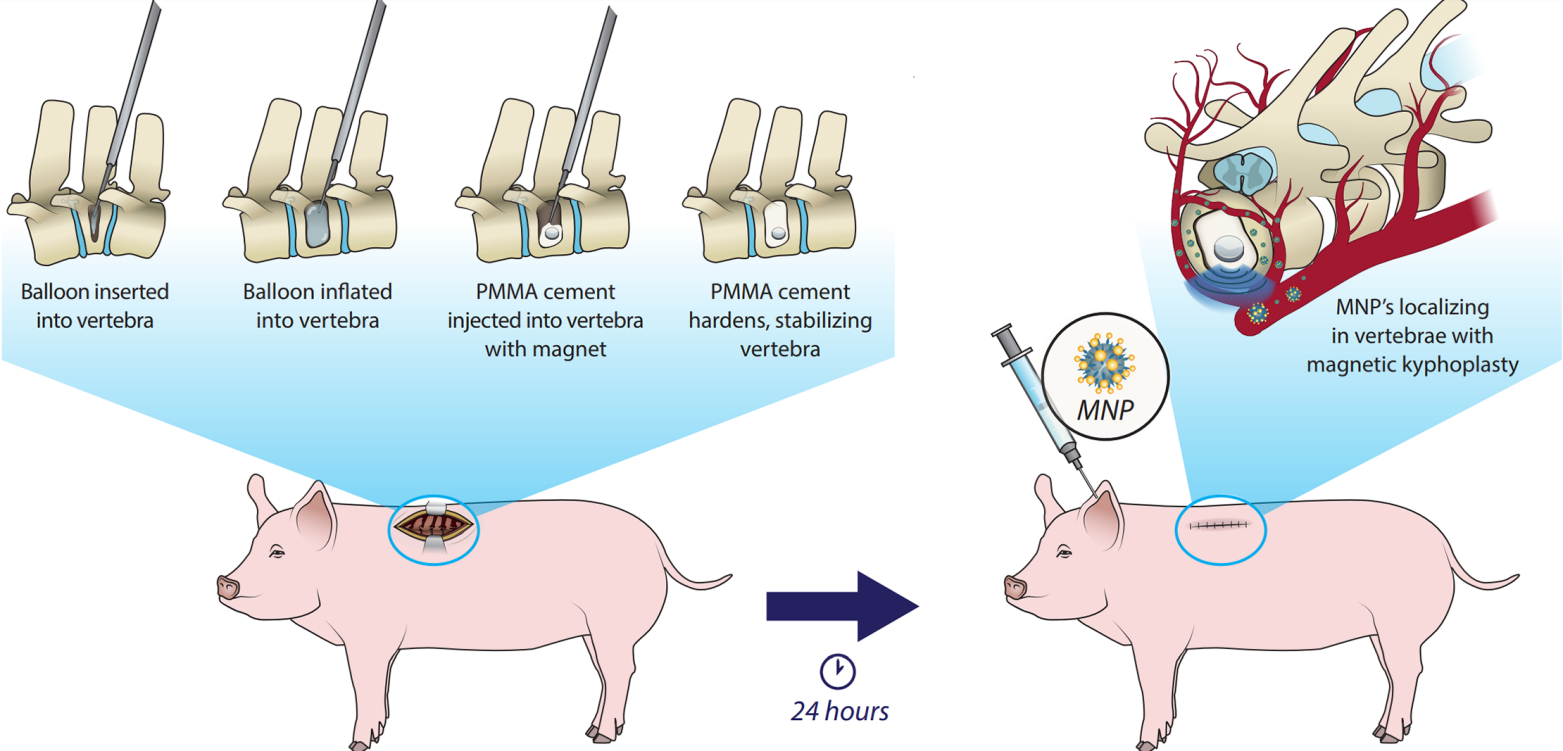
Schematic representation of experimental outline used to establish a porcine cement kyphoplasty model. In this experimental setup, a balloon is inserted into a porcine vertebra. Following inflation of the balloon, PMMA cement with or without magnets is injected into the vertebra. 24-hours after surgery, magnetic nanoparticles (MNPs) are injected systemically via the ear vein. (Image illustrated by Victoria Zakrzewski.).

**Fig 2:**
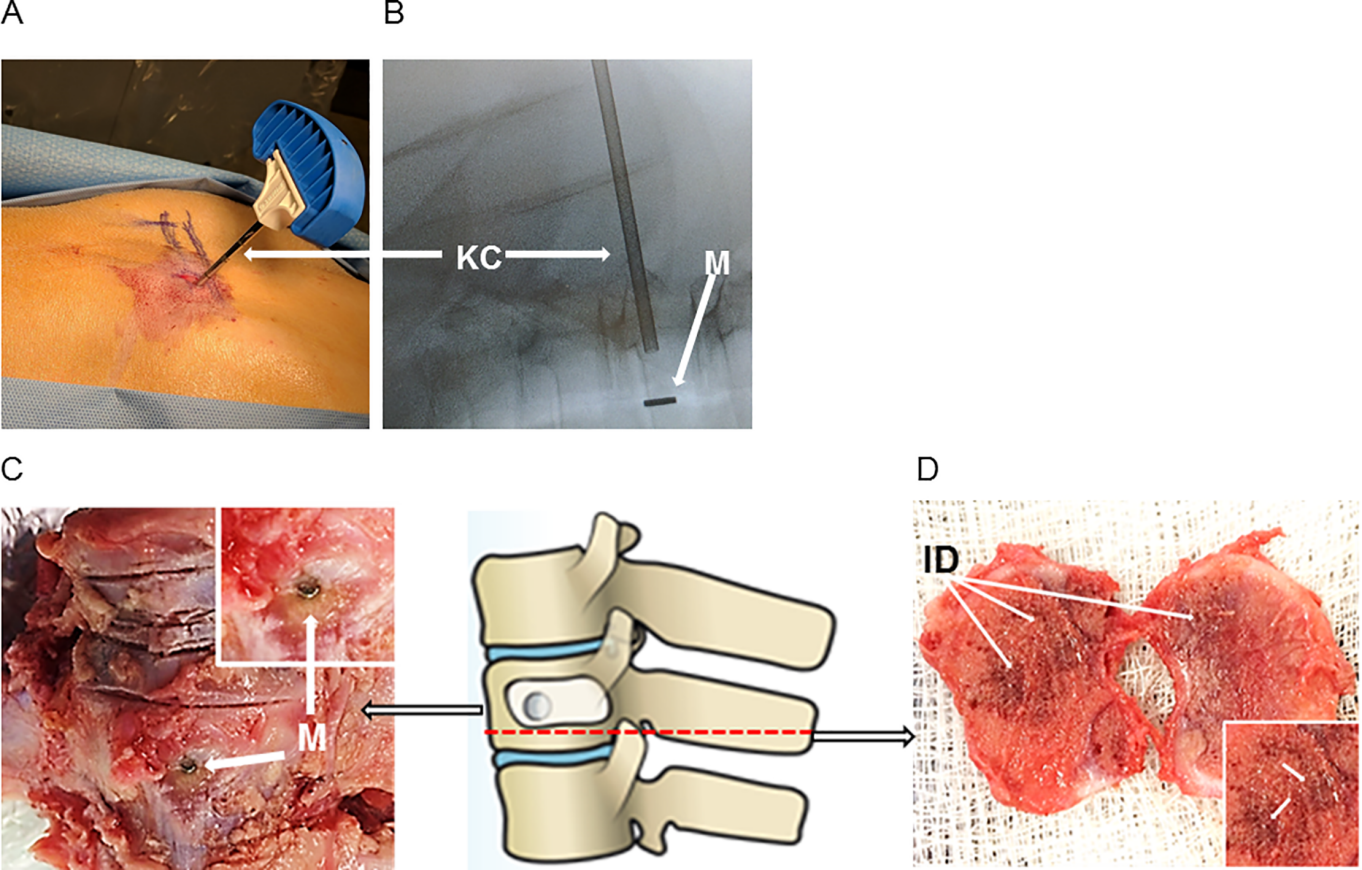
Successful establishment of a porcine magnetic kyphoplasty model. (**A**) Magnetically enhanced kyphoplasty cement was injected into the vertebrae of a male pig via a kyphoplasty catheter (KC). (**B**) Intraoperative X-ray of the experimental vertebra showing proper alignment of the KC and placement of the cement and magnet (M) within the vertebral body. (**C**) The magnets introduced during kyphoplasty were seen upon examination of the dissected experimental vertebral body. (**D**) Sectioning the vertebrae of the magnetic kyphoplasty reveals a darker coloration of the bone marrow within the experimental vertebral body as a result of magnetic nanoparticle iron deposition (ID).

To study the novel vertebral drug delivery system, we developed a porcine kyphoplasty model (Fig 1). First, the Jamshidi cannulated needles are inserted into the vertebral body through the pedicles bilaterally (Fig 2A), allowing inflatable tamps to be used to compress the surrounding bone. Following deflation and removal of the tamps, polymethylmethacrylate (PMMA) cement mixed with neodymium-iron-boron (NdFeB) magnets were implanted into the thoracic vertebral body. Gas ethylene oxide sterilized NdFeB magnets were used, as they are known to be biocompatible [21]. As an internal control, PMMA without magnets was placed two vertebrae levels below the magnet enhanced kyphoplasty. Intraoperative X-ray used for guidance demonstrated that the magnets were successfully inserted into an upper thoracic vertebral body (Fig 2B). The cement mixture was allowed to harden and set, then the incision was closed, and the pig allowed to recover. As kyphoplasty is minimally invasive and pain management was provided, the male pig recovered well from the surgery with no signs of complication. 24 hours later, a solution of magnetic nanoparticles (MNPs) was infused through the ear vein without any signs of hyperacute infusion reaction. We hypothesized that the introduction of the magnet into the vertebral body would provide sufficient localizing magnetic signal for systemically injected magnetic nanoparticles (MNPs).

The thoracic vertebral bodies that were subjected to kyphoplasty with and without magnets along with a control (no kyphoplasty) lumbar vertebra were removed from the animal 24 hours after MNP infusion. Close examination of the exterior of the vertebral bodies reveals a dark spot on one of the bodies indicating the placed magnet during the kyphoplasty (Fig 2C), confirming the intraoperative X-ray results. Inspection of the lumbar vertebra and the thoracic vertebra that did not have a magnet placed did not display any similar dark spots. The darker color of the magnet enhanced kyphoplasty bone marrow resulted from deposition of the MNPs, whose iron core confers a dark color (Fig 2D).

### Localization of MNPs in magnetic kyphoplasty

**Fig 3:**
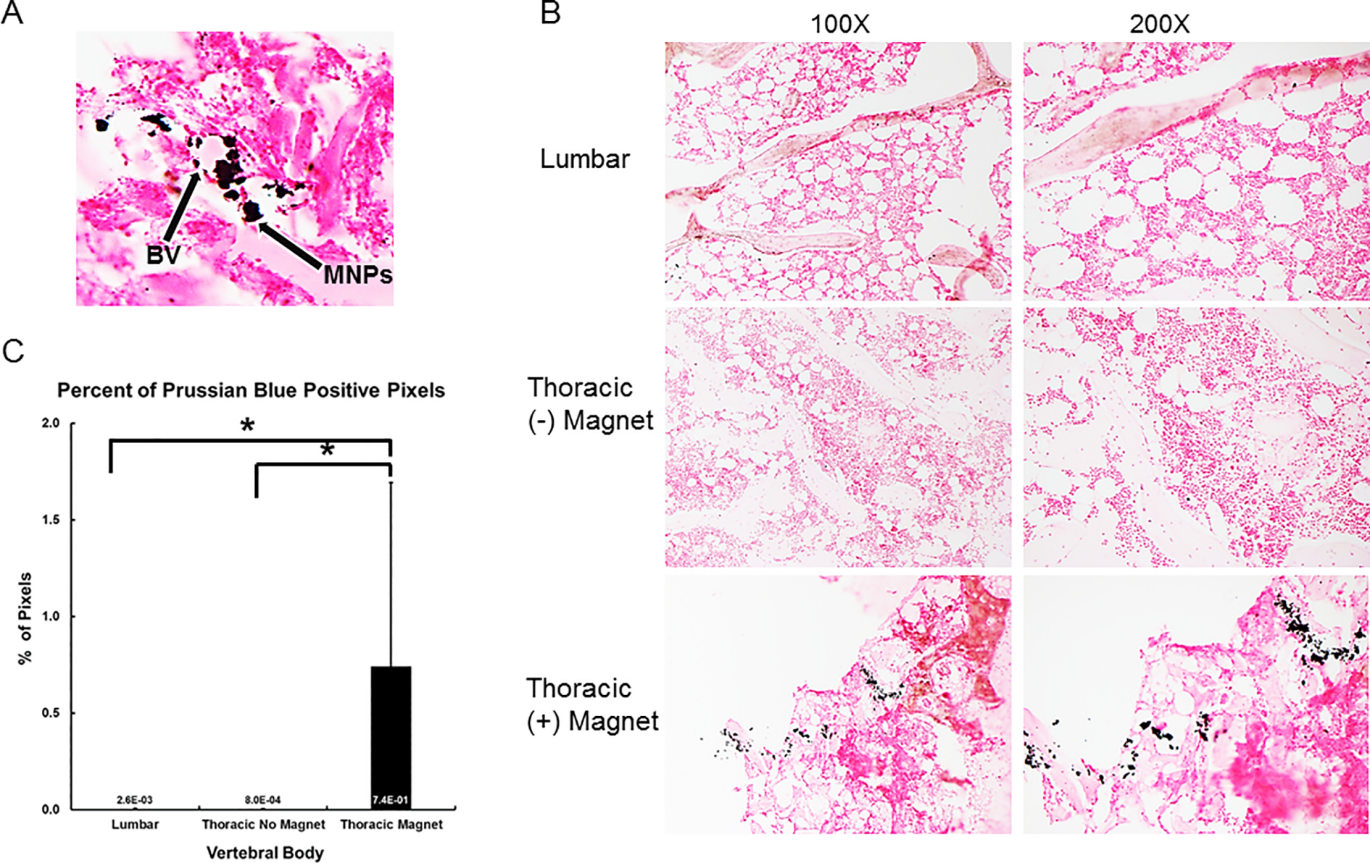
Magnetic nanoparticle localization and quantification in thoracic vertebra. (**A**) Prussian blue staining of histological sections from thoracic vertebra injected with magnetic cement display heavy concentrations of magnetic nanoparticle (MNP) clusters near the blood vessels (BV), with diffuse MNPs throughout the tissue, indicating the MNPs can exit the blood vessel lumen and enter the bone marrow space. (**B**) No MNPs were noted in lumbar vertebrae or thoracic vertebra that did not have a magnet. (**C**) Quantification of Prussian blue staining in 10 fields of view of each experimental group. * Compared with thoracic vertebra containing magnets, p<0.05.

Histological staining using Prussian blue was used to further visualize the presence or absence of MNPs. Paraffin embedded, 12μm sections of vertebral bodies were exposed to Prussian blue and nuclear fast red stains. Prussian blue stain labeled the MNPs, as they have an iron core, and nuclear fast red allowed for visualization of tissue structure. The magnet-enhanced kyphoplasty displayed typical bone marrow structure and exhibited large clustering of the Prussian blue stain in addition to a diffuse spread of Prussian blue labeled particles throughout the tissue (Fig 3A and 3B). Further inspection at higher magnifications revealed that the large MNP clusters occurred perivascularly, and that the diffuse staining was distributed throughout the tissue away from the blood vessels (Fig 3A).

Comparison of the microscopic images of the magnet-enhanced kyphoplasty vertebral bodies to the control kyphoplasty thoracic vertebrae and the lumbar vertebrae (no surgery) revealed marked differences (Fig 3B). In contrast to the magnetic kyphoplasty vertebrae, the thoracic control kyphoplasty displayed typical structure and very little, if any, Prussian blue staining. The lumbar (no surgery) bone marrow appeared very similarly, displaying characteristic cellular architecture with little to no Prussian blue stain (Fig 3B). Ultimately, the histological staining revealed the presence of MNPs in large quantities within the magnet-enhanced kyphoplasty vertebral body, and relative absence of MNPs within the control kyphoplasty and non-operated lumbar vertebral bodies. These qualitative observations were further quantified using pixel analysis of the Prussian blue data. The number of Prussian blue positive pixels were determined using ImageJ and used to calculate the percentage of pixels which displayed Prussian blue stain. The magnetically enhanced vertebral body had a statistically significant higher number of Prussian blue positive cells compared to control sample (Fig 3C).

## Discussion

Although kyphoplasty can provide symptomatic support, it does not address the underlying causes of VCF. Here, we provide a proof of concept demonstration of a novel, innovative drug delivery system for the spinal column that utilizes magnet enhanced kyphoplasty to target systemically delivered magnetic nanoparticles to the vertebral body. We successfully developed the magnetic kyphoplasty procedure, the first of its kind, that combines the palliative care of kyphoplasty with the ability to target systemically delivered magnetic nanoparticles to the vertebral body. Moreover, our model system was performed in a Landrace-Yorkshire pig, which demonstrates the ability of our approach to work in large animals. The animal tolerated the procedure and recovered with no complications. A lack of a hyperacute infusion reaction to the MNPs further supports the safety of this procedure. In order to demonstrate the ability of intravertebral magnets to localize MNPs, the thoracic magnet-enhanced vertebra was compared to both the thoracic control kyphoplasty and non-operative lumbar vertebrae after systemic infusion of the MNPs. Both macroscopic and histologic quantification demonstrated increased ability of magnet-enhanced kyphoplasty to localize magnetic nanoparticles to the vertebral body compared to standard kyphoplasty. The bone marrow of both vertebral bodies displayed characteristic structure under microscopy, indicating the addition of magnets during kyphoplasty does not appear to negatively affect the tissue more than typical kyphoplasty procedures. The Prussian blue staining pattern displayed large perivascular clusters and diffuse individual MNPs throughout the bone marrow space, indicating the MNPs can exit the blood vessel lumen and spread throughout the vertebral body tissue. In their entirety, the results demonstrate that our novel procedure of magnet-enhanced kyphoplasty can be utilized to successfully localize magnetic nanoparticles to the vertebral body. With further development, magnet-enhanced kyphoplasty has the potential to become an integrated surgical option for primary and metastatic spinal column tumor patients that combines the spinal stabilization and pain relief provided by traditional kyphoplasty with targeted chemotherapy delivery.

The results signify that this proof of concept for the localization of MNPs to the vertebral body via magnet-enhanced kyphoplasty. This novel technique has potential for treatment and localized drug delivery for spinal fractures in osteoporotic patients as well as spinal column cancer patients as the magnetic nanoparticles can be loaded with different pharmacological agents. For instance, magnetic nanoparticles could be loaded with bisphosphonates or bone morphogenic protein in an attempt to treat the underlying osteoporosis that caused the VCF. For neoplastic causes of VCFs, the particles could be loaded with the chemotherapeutic of choice. In addition, MNPs with different pharmacological agents could be used in the same patient to increase efficacy of treatment and prevent subpopulations of the tumor from developing chemoresistance. By demonstrating that our novel magnetic kyphoplasty can selectively deliver MNPs to the vertebral body, efficacy studies of the many therapeutic uses can to be conducted. However, there are limitations to this study, such as the limited sample size. Also, although the platform can deliver MNPs to the vertebral body, this experiment did not formally demonstrate that MNPs carrying drug cargos can be delivered to the vertebral body. Additional animal studies are needed to address the efficacy and long-term effects of our novel delivery system. Development of an advanced metastatic pig model, such as prostate cancer, will enable future studies to investigate the ability of our delivery system to treat spinal column lesions. Regardless of the limitations, this experiment demonstrates that magnet-enhanced kyphoplasty is able to localize systemically-injected magnetic nanoparticles to specific locations in the spinal column.

The results from this study propose a novel method that integrates kyphoplasty and a magnetically localizable drug delivery system to target drug loaded magnetic nanoparticles to the spinal column. Investigations for this proposed proof-of-concept were successfully carried out on a pig model. In a comparison between the experimental vertebral body that received PMMA and magnets and the control vertebral body that only received PMMA or a lumbar vertebra, stark differences were observed in the gross and microscopic observations following intravenous injection of MNPs. Given that both the controls and experimental vertebra of interest came from the same pig, the localization of the systemically-injected magnetic nanoparticles to the magnet-enhanced experimental vertebrae provides proof that magnet-enhanced kyphoplasty can be utilized as a means of localizing magnetic nanoparticles to targeted areas in the spinal column. This novel kyphoplasty procedure has the potential to merge two separate, extremely effective treatments into one cohesive therapy for patients with VCFs to provide a means of improving quality of life and long-term prognosis.
